# Early detection of amniotic fluid embolism leading to extracorporeal membrane oxygenation prior to cardiac arrest

**DOI:** 10.21542/gcsp.2024.51

**Published:** 2024-12-31

**Authors:** Enad Haddad, Hamza Muhammadzai, Shayan Khan, Angad Bedi, Rajeshkumar Patel

**Affiliations:** 1 Department of Internal Medicine, Jefferson Abington Hospital, Abington, Pennsylvania, USA; 2 Department of Cardiology, Thomas Jefferson University Hospital, Philadelphia, Pennsylvania, USA; 3 Abington Pulmonary and Critical Care Associates, Jefferson Abington Hospital, Abington, Pennsylvania, USA

## Abstract

Amniotic fluid embolism is a life-threatening peripartum condition with high morbidity and mortality rates. It is defined as the passage of fetal material into the maternal circulation which elicits a multisystem reaction that can lead to disseminated intravascular coagulopathy and severe hemodynamic instability. Despite established diagnostic criteria for amniotic fluid embolism, its identification relies heavily on clinical judgment, and early diagnosis is key to patient survival. In several cases, early utilization of extracorporeal membrane oxygenation support has provided a salvage approach to correct cardiopulmonary collapse. Herein, we present a case of successful application of venoarterial extracorporeal membrane oxygenation in a 29-year-old woman who experienced refractory respiratory distress and hemodynamic instability secondary to amniotic fluid embolism following vaginal delivery. Improving diagnostic protocols and optimizing the utilization of extracorporeal membrane oxygenation is essential to improve outcomes in this high-risk obstetric emergency.

## Introduction

Amniotic fluid embolism (AFE) is a rare, yet potentially fatal condition with a mortality rate of up to 60%^1^. It occurs when the maternal-fetal barrier is compromised, leading to the passage of fetal material into the maternal circulation, eliciting a multisystem reaction^2^. Treatment is focused on avoiding or reversing cardiopulmonary collapse. Extracorporeal membrane oxygenation (ECMO) has been successfully implemented in previous AFE cases for advanced hemodynamic support. We present a case of a 29-year-old female who experienced hemodynamic collapse 30 min postpartum secondary to AFE, which was successfully managed with venoarterial ECMO.

**Figure 1.**
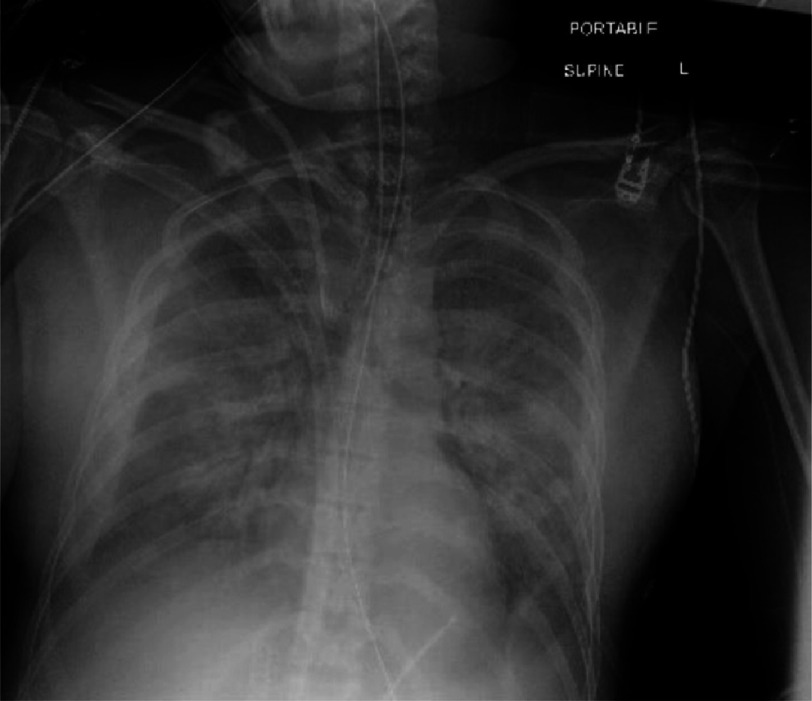
Chest X-ray showing signs of pulmonary edema.

## Case presentation

A 29-year-old nulliparous woman with no significant medical history was admitted for elective induction of labor with oxytocin at 40 weeks and 4 days gestation. Following a relatively brief four-hour labor, she delivered a healthy male baby vaginally. However, within 30 min post-delivery, the patient experienced a sudden onset of sinus tachycardia and shortness of breath requiring noninvasive positive pressure ventilation. X-ray of the chest revealed possible pulmonary edema (Figure 1) and possible acute respiratory distress syndrome. With persistently increased oxygen requirements, the patient was intubated and mechanically ventilated. However, despite being on 100% fraction of inspired oxygen and a peak end-expiratory pressure of 10 mmHg, the patient remained hypoxic and hypotensive. Stress dose steroids, epinephrine, and vasopressin were commenced, improving blood pressure.

**Figure 2.**
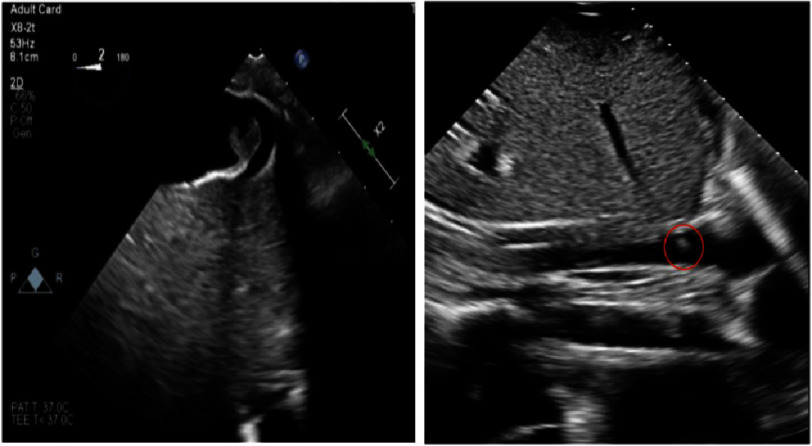
(a, b) Transthoracic echocardiography showing a mobile mass measuring 10 mm × 7 mm just distal to the hepatic vein.

**Figure 3.**
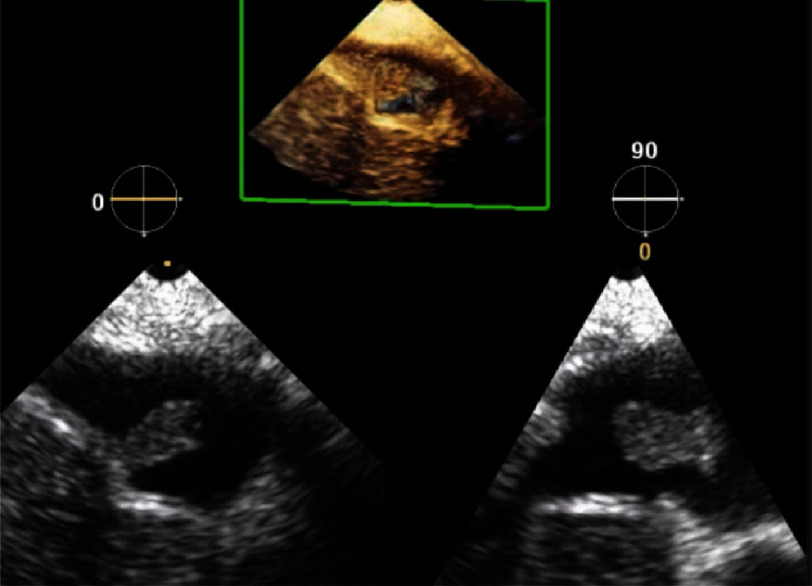
Intraoperative transesophageal echocardiography and 3D image of the amniotic fluid embolism.

A bedside echocardiogram showed moderately decreased left ventricular systolic function with global hypokinesis, with moderately to severely decreased right ventricular function, along with inferior vena cava dilation, with a mobile mass measuring 10 mm x 7 mm just distal to the hepatic vein (Figure 2a, Figure 2b, and Figure 3). A decision was made to perform emergent bedside placement of venoarterial ECMO *via* femoral artery and femoral vein. Heparin therapy was started as well. Lab workup showed a white blood cell count of 36,000 (4,500 to 11,000 per microliter), D-Dimer of 61,999 (normal <500 ng/mL), and fibrinogen of 107 (normal 200–400 mg/dL), concerning for disseminated intravascular coagulation (DIC) secondary to AFE. Massive transfusion protocol was activated and the patient received 5 units of packed red blood cells, 4 units of fresh frozen plasma (FFP), and 20 units of cryoprecipitate.

Complications ensued. The patient experienced abdominal distention and further hemodynamic instability. She was found to have injury to the right common femoral artery (CFA) from the ECMO cannula, and right CFA repair with a patch was performed.

Despite the challenges and after six days on ECMO support, she was successfully decannulated and extubated on the seventh day. Heparin therapy was completed in accordance with ECMO protocol. The patient was eventually discharged in a stable condition for acute rehabilitation.

## Discussion

AFE is characterized by the disruption of the maternal-fetal interface, with passage of amniotic fluid to the maternal circulation^3^. It is hypothesized that it leads to unregulated activation of proinflammatory and vasoactive cytokines which lead to a plethora of life-threatening pathologies. These include disseminated intravascular coagulation (DIC), acute respiratory failure with severe hypoxemia, acute right heart failure, and eventual late onset left ventricular failure^1,3^. Furthermore, pathophysiological analysis of AFE revealed that it also has a component of septic and anaphylactic shock in its pathophysiology^4^. It usually presents with a sudden onset of dyspnea, hypotension that is not proportionate to the amount of blood lost, and hemodynamic compromise that is usually quickly followed by cardiopulmonary arrest^5^. Risk factors include maternal age above 35 years, multiple pregnancies, medical induction of labor, placenta previa, placenta accreta, uterine rupture, eclampsia, polyhydramnios, and multiple pregnancy^4,6^.

The Society of Maternal-Fetal Medicine and the Amniotic Fluid Embolism Foundation have proposed four criteria for AFE diagnosis^7^:

1. Acute cardiac arrest or both respiratory and hemodynamic collapse
2. DIC
3. The absence of fever
4. The clinical onset being during labor or within 30 min of delivery

The patient in the case we presented successfully met all these criteria to be diagnosed with AFE. Additionally, her risk factors included medical induction of labor.

Early detection of AFE is crucial, and entails recognizing the most common clinical and diagnostic components of its presentation, particularly given its low incidence. The most common signs and symptoms include hypotension, respiratory distress, and cyanosis^8^. The simultaneous occurrence of the four diagnostic criteria mentioned above is rare in most differential diagnoses, increasing suspicion for AFE in that case^9^. In terms of laboratory workup, there are a few serum markers that could potentially be diagnostic but have not been studied on larger scales. These include zinc coproporphyrin, sialyl Tn antigen, and C3 and C4 complement^8^. Additionally, the detection of amniotic fluid tissue elements in maternal pulmonary arterial circulation was previously studied but eventually deemed to be of low diagnostic value^9^. Echocardiography findings can reflect the biphasic hemodynamic response to AFE, where an initial increase in pulmonary vascular resistance and right ventricular failure are followed by left ventricular failure^10^. However, diagnosing AFE remains heavily reliant on excluding other differential diagnoses, and early specific diagnostic indicators are yet to be established.

In terms of treatment, the goal is to achieve rapid maternal cardiopulmonary stabilization during anaphylactic shock, preventing persistent hypoxemia and end-organ failure, and ECMO can quickly provide cardiopulmonary support and minimize these complications. ECMO is an essential tool that can replace cardiopulmonary function temporarily to ensure adequate oxygenation and decrease cardiac oxygen consumption. It has been shown to be of significant benefit in several cases in patients with cardiopulmonary and hemodynamic collapse in patients with AFE. Its prompt initiation should always be considered given the significantly high rate of mortality in AFE, with 50% of deaths occurring within the first hour after diagnosis, and 25–50% occurring in the following 4–5 h^5^.

Indications to initiate ECMO include^5^:

1. Severe acute respiratory failure with an expected mortality rate of >80%
2. Reversible respiratory failure
3. Failure of return of spontaneous circulation in cardiac arrest
4. Inability to maintain SBP above 70 mmHg.

In regard to ECMO duration, previous studies demonstrated the need of ECMO use up to 150 h, while some had patients weaned off ECMO within 4 h only. This difference might be explained by the commencement of ECMO before the occurrence of myocardial damage. Additionally, the risk of hemolysis, bleeding, and renal impairment associated with ECMO makes timely weaning crucial to prevent further complications^5^.

Ultimately, ECMO is an essential life-saving intervention that has improved outcomes in patients with AFE. Because of the high rate of mortality, and the serious neurological and other end-organ complications in survivors, it was crucial in our case to initiate ECMO support promptly. Our patient successfully recovered without any deficiencies in her neurological function.

## What have we learned?

AFE does not have a unique clinical picture, yet timely diagnosis and treatment initiation are crucial for survival given its high fatality rate. ECMO has proven effective as a tool to correct patients’ cardiopulmonary compromise. Further research is necessary for the early diagnosis of AFE and to determine the practices for initiating and weaning ECMO in patients with AFE.

## Funding

This research has not received any specific grant from public, commercial, or non-profit sector agencies.

## Conflicts of Interest

The authors have no conflicts of interest to declare

## Author Contributions

**Conceptualization**: Enad Haddad, Hamza Muhammadzai, Shayan I. Khan, Angad Bedi, and Rajeshkumar Patel.

**Writing - original draft**: Enad Haddad.

**Writing - review & editing**: Enad Haddad, Hamza Muhammadzai, Shayan I. Khan, Angad Bedi, and Rajeshkumar Patel.
